# Six novel immunoglobulin genes as biomarkers for better prognosis in triple-negative breast cancer by gene co-expression network analysis

**DOI:** 10.1038/s41598-019-40826-w

**Published:** 2019-03-14

**Authors:** Huan-Ming Hsu, Chi-Ming Chu, Yu-Jia Chang, Jyh-Cherng Yu, Chien-Ting Chen, Chen-En Jian, Chia-Yi Lee, Yueh-Tao Chiang, Chi-Wen Chang, Yu-Tien Chang

**Affiliations:** 1 Graduate of Medical Sciences, National Defense Medical Center, Taipei, Taiwan; 2 Department of Surgery, Songshan Branch of Tri-Service General Hospital, National Defense Medical Center, Taipei, Taiwan; 3 Division of Biostatistics and Informatics, Department of Epidemiology, School of Public Health, National Defense Medical Center, Taipei, Taiwan; 4 Big Data Research Center, Fu-Jen Catholic University, New Taipei City, Taiwan; 5 Department of Public Health, China Medical University, Taichung, Taiwan; 6 Department of Public Health, College of Health Sciences, Kaohsiung Medical University, Kaohsiung, Taiwan; 7 Department of Healthcare Administration and Medical Informatics, College of Health Sciences, Kaohsiung Medical University, Kaohsiung, Taiwan; 8 Department of Medical Research, Kaohsiung Medical University Hospital, Kaohsiung City, Taiwan; 9 Graduate Institute of Clinical Medicine, College of Medicine, Taipei Medical University, Taipei, Taiwan; 10 Division of General Surgery, Department of Surgery, Tri-Service General Hospital, National Defense Medical Center, Taipei, Taiwan; 11 School of Nursing, College of Medicine, Chang Gung University, Taoyuan, Taiwan; 12 Department of Pediatrics, Chang Gung Memorial Hospital, Taoyuan, Taiwan

## Abstract

Gene co-expression network analysis (GCNA) can detect alterations in regulatory activities in case/control comparisons. We propose a framework to detect novel genes and networks for predicting breast cancer recurrence. Thirty-four prognosis candidate genes were selected based on a literature review. Four Gene Expression Omnibus Series (GSE) microarray datasets (n = 920) were used to create gene co-expression networks based on these candidates. We applied the framework to four comparison groups according to node (+/−) and recurrence (+/−). We identified a sub-network containing two candidate genes (*LST1* and *IGHM*) and six novel genes (*IGHA1*, *IGHD*, *IGHG1*, *IGHG3*, *IGLC2*, and *IGLJ3*) related to B cell-specific immunoglobulin. These novel genes were correlated with recurrence under the control of node status and were found to function as tumor suppressors; higher mRNA expression indicated a lower risk of recurrence (hazard ratio, HR = 0.87, p = 0.001). We created an immune index score by performing principle component analysis and divided the genes into low and high groups. This discrete index significantly predicted relapse-free survival (RFS) (high: HR = 0.77, p = 0.019; low: control). Public tool KM Plotter and TCGA-BRCA gene expression data were used to validate. We confirmed these genes are correlated with RFS and distal metastasis-free survival (DMFS) in triple-negative breast cancer (TNBC) and general breast cancer.

## Introduction

Breast cancer (BC) is perhaps the most well-studied malignancy in the world. Approximately 1.7 million women were diagnosed with the disease in 2012, making it a global priority^1^. There is an urgent need to identify risk factors associated with recurrence to address this serious problem^2^.

Microarray analysis has contributed to our understanding of the heterogeneity and complexity of BC^3^, and it has enabled the identification of gene signatures for diagnosis, molecular characterization, prognosis prediction and treatment recommendation^4–6^. Networks of topological characteristics can potentially serve as predictive biomarkers through network-based classification^7,8^, and the topology of biological networks has increasingly been used to complement studies of individual genes or gene sets^9,10^. Several gene network analysis tools based on various methodologies have been developed, including GeneMania^11^, BisoGenet^12^, Cytoscape^13^, and DAVID^14^.

Gene co-expression network analysis (GCNA) provides insight into novel biological mechanisms and is complementary to standard differential expression (DE) analysis. This method has proven to be an attractive and effective tool for understanding BC^10,15–17^. However, gene co-expression networks (GCN) from single transcriptomic studies are often less informative and generalizable due to cohort bias and a limited sample size, whereas the use of integrated analysis through the combination of multiple transcriptomic studies provides more accurate and comprehensive results^18^. Therefore, we applied GCNA and integrated microarray analysis, and we considered candidate genes related to BC prognosis to design an analysis procedure and to investigate novel genes and networks related to BC recurrence.

## Results

**Figure 1:**
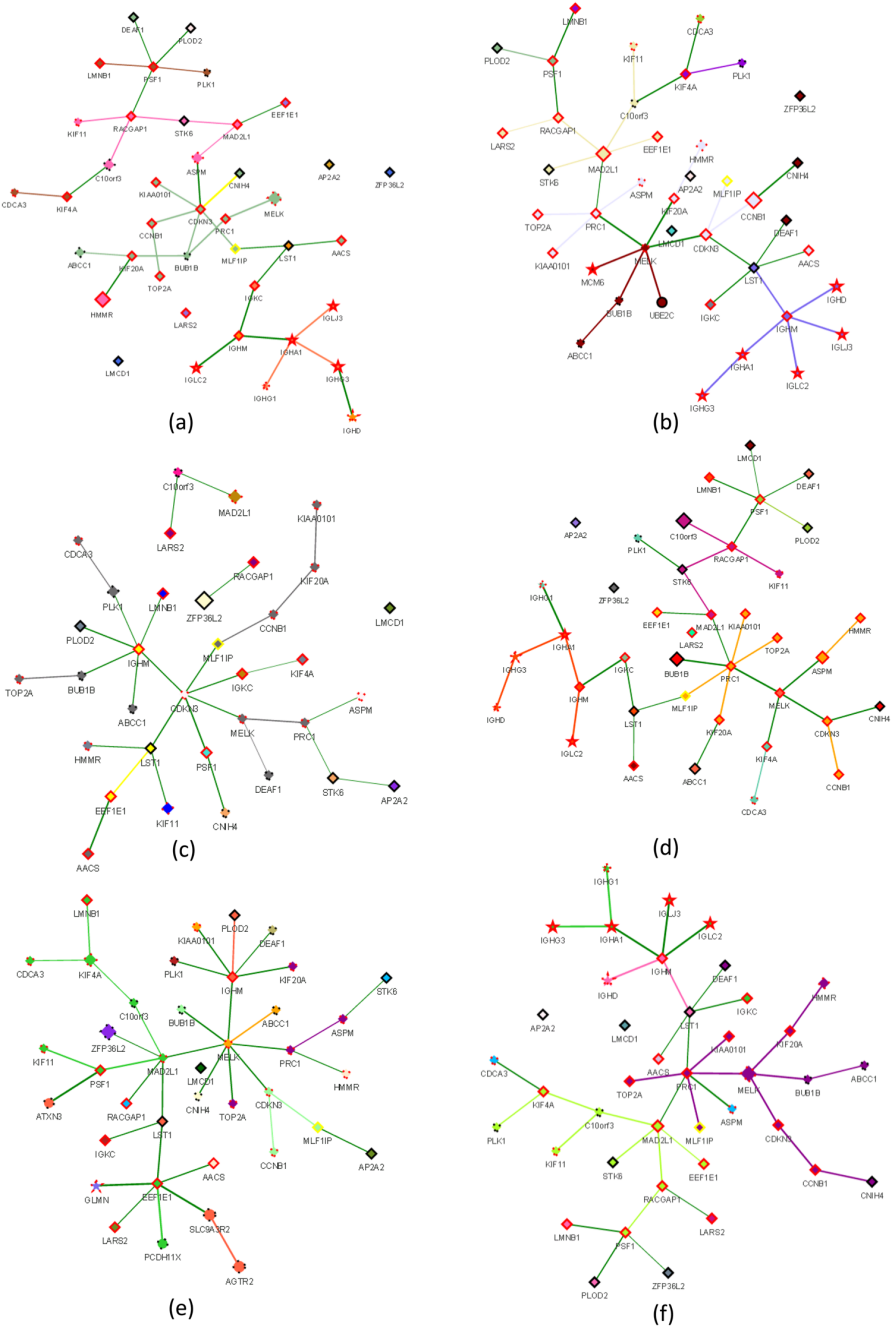
Gene co-expression networks. Co-expression networks of six subgroups, (**a**) Recurrence (+), (**b**) Recurrence (−), (**c**) Node (+) and Recurrence (+), (**d**) Node (+) and Recurrence (−), (**e**) Node (−) and Recurrence (+), (**f**) Node (−) and Recurrence (−). The width of the gene connection indicates the degree of correlation between genes. Colors of the gene icons and connecting lines denote similar gene expression patterns for genes in the same color, which were analyzed by hierarchical clustering. Connection lines in green denote neighboring genes that do not belong to the same cluster. Size of the gene icon reflects the absolute value of cv of gene expression. The 34 candidate genes are represented by diamonds; co-expressed genes are represented by circles, and significant recurrence associated co-expressed genes are represented by stars. The gene icon frame is shown in red if 0.01 ≤ p < 0.05, and it is shown in yellow if p < 0.01. Up-regulated genes are shown by a dashed line, whereas down-regulated genes are shown by a solid line.

We made comparisons between groups using GCNA with r > 0.9 and edge limit = 1. Comparison networks between cases of recurrence and no recurrence were generated, and *UBE2C*, *MCM6* and *IGHG1* were found to be highly differentially co-expressed genes. (Table 1 and Fig. 1) Common genes in the two networks were *IGHA1*, *IGHD*, *IGHG3*, *IGLC2*, and *IGLJ3*. Highly co-expressed genes in each of the four comparison groups classified by node (+/−) and recurrence (+/−) are shown in Table 2 and Fig. 1. Regardless of node status, highly co-expressed genes within the network of no recurrence were *IGHA1*, *IGHD*, *IGHG1*, *IGHG3*, *IGLC2*, and *IGLJ3*. Cox proportional hazard ratio regression analysis found these genes to be significantly correlated with the recurrence of BC, regardless of node status (Table 3, Fig. 1). Logistic regression analysis revealed a significant correlation with node status, with an odds ratio (OR) range of 2.3–23 (p < 0.001). These six highly co-expressed genes for *LST1* and *IGHM* belong to a cluster and are related to immune function (Fig. 1).

**Table 1:** Highly co-expressed genes correlated with BC recurrence.

Recurrence	Non-recurrence
IGHA1*	IGHA1*
IGHD*	IGHD*
IGHG1*	
IGHG3*	IGHG3*
IGLC2*	IGLC2*
IGLJ3*	IGLJ3*
	MCM6*
	*UBE2C*

P values were calculated using Cox proportional hazard ratio regression for breast cancer recurrence controlled by node (+/−) and *means p value < 0.05.

**Table 2:** Highly co-expressed genes correlated with BC recurrence grouped by node status.

Group	Recurrence	Non-recurrence
Node+	None	IGHA1*
		IGHD*
		*IGHG1*
		IGHG3*
		IGLC2*
		IGLJ3*
Node−	*AGTR2*	IGHA1*
	*ATXN3*	IGHD*
	GLMN**	*IGHG1*
	*PCDH11X*	IGHG3*
	*SLC9A3R2*	IGLC2*

P values were calculated using Cox proportional hazard ratio regression for breast cancer recurrence controlled by node (+/−). *Means p value < 0.05, **means p value < 0.01.

**Table 3:** Univariable Cox proportional hazard ratio regression of novel co-expressed genes for breast cancer recurrence.

Gene	B	HR	P
IGHA1*	−0.17	0.85	0.02
IGHD*	−0.13	0.87	0.03
IGHG1*	−0.24	0.78	0.02
IGHG3*	−0.17	0.84	0.02
IGLC2*	−0.16	0.86	0.01
IGLJ3*	−0.16	0.85	0.02
MCM6*	0.22	1.24	0.02
*UBE2C*	0.10	1.10	0.13
*AGTR2*	−0.04	0.96	0.50
*ATXN3*	−0.02	0.98	0.81
GLMN*	0.30	1.35	0.05
*PCDH11X*	−0.03	0.97	0.65
*SLC9A3R2*	0.02	1.02	0.79

P values (p) were calculated using Cox proportional hazard ratio regression for breast cancer recurrence controlled by node (+/−). *Means p < 0.05. HR: hazard ratio, B: the coefficient of predictors in the Cox proportional hazard ratio regression.

Recently, studies have found that robust levels of tumor-infiltrating lymphocytes (TILs) are associated with increased disease-free survival (DFS) and overall survival (OS) rates in triple-negative breast cancer (TNBC) patients with and without treatment. There have also been efforts to develop a standardized methodology for evaluating TILs^19^. Their presence at diagnosis is associated with a pathologic response to neoadjuvant therapy as well as increased DFS and OS following adjuvant chemotherapy in certain subtypes^20,21^.

**Figure 2:**
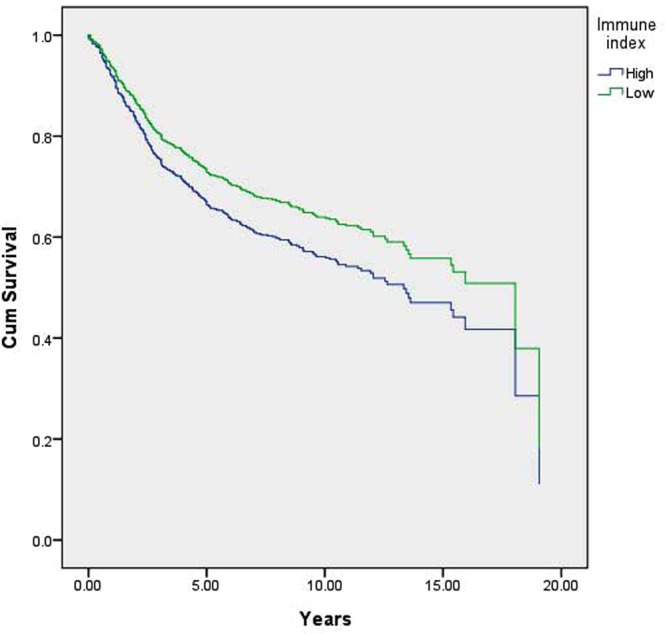
Cox proportional hazard ratio regression prediction model of a relapse-free survival curve based on the immune index.

We speculate that highly co-expressed immune-related genes can be used for the prognosis and treatment of general BC as well as TNBC, and we have established an immune response index to explore the relationships between specific genes and BC recurrence. Because these six genes were highly correlated, they were replaced with a component score by using principle component analysis (PCA). This score had a value range of −1.93~ 1.83 (mean = 0, sd = 1). Cox proportional hazard ratio regression analysis (under the control of node status) was also performed to investigate the impact of the component score on recurrence. It was found that the risk of recurrence was reduced by approximately 13% (HR = 0.87, p = 0.014) for each additional unit of the component score (Table 4). In addition, to divide the samples into high and low immune index groups, we used the 40th percentile (value: −0.5) of the component score as the group’s cut-off point. Using the low group as the control, the immune index effectively distinguished recurrence status, with a hazard ratio (HR) of 0.774 (p = 0.019). (Table 4, Fig. 2).

**Table 4:** Cox proportional hazard ratio regression of the immune index for breast cancer recurrence.

Model 1^a^	B	HR	P
Node	0.53	1.70	0.001
Immune index	−0.14	0.87	0.014
**Model 2** ^**b**^			
Node	0.461	1.586	0.004
Low immune index (n = 355)			ref
High immune index (n = 552)	−0.256	0.774	0.019

^a^Immune index used in this model is a continuous variable.

^b^Immune index in this model was divided into high and low immune index groups by cutoff point −0.5.

B: the coefficients of predictors, HR: hazard ratio and ref: reference group in the Cox proportional hazard ratio regression.

Since TNBC is an aggressive subtype and difficult to treat, we wanted to know whether these immune-related genes are correlated by using KM Plotter online cancer survival analysis tool (http://kmplot.com/analysis/)^22^. The TNBC validation data sets consist of 255 RFS and 43 DMFS cases. All immunoglobulin-related genes were significantly associated with RFS and DMFS with the exception that IGHD was not related to RFS (Table 5).

**Table 5:** The validation of immune-related genes using TNBC samples from KM Plotter online cancer survival analysis tool (http://kmplot.com/analysis/).

RFS	DMFS
**(a) IGLC2**	
	
**(b) IGHM**	
	
**(c) IGHD**	
	
**(d) IGHA1**	
	
**(e) IGLJ3**	
	
**(f) LST1**	
	
**(g) IGHG1**	
	
**(h) IGHG3**	
	

In order to validate these genes in a larger BC data set, we used TCGA-BRCA gene expression data sets representing 1,215 tumors (Supplementary Fig. 1). DMFS (n = 68) and RFS (TNBC (+): n = 131; TNBC (−): n = 637) samples were selected and stratified by TNBC status (negative/positive) (Supplementary Table 1). All of these BC patients had received initial treatment. Because there are no corresponding gene symbols for our six immunoglobulin-related genes in TCGA-BRCA microarrays, we validated all related immunoglobulin genes: IGLL3, IGLL1, IGSF9B, IGDCC3, IGDCC4, IGBP1, IGSF5, IGSF11, IGSF22, IGSF21, IGHMBP2, IGSF10, IGSF8, IGSF9, IGSF6, IGSF1, IGSF3, IGFN1, and IGJ. Stage, TNM stage, PR status and node status were significantly associated with RFS and DMFS in the univariable Cox proportional-hazards regression models (Supplementary Table 2). The clinical variables were analyzed with the immunoglobulin-related genes by using multivariable Cox proportional-hazards regression. Only node status was found to be significantly related to recurrence with biomarkers in the multivariable Cox proportional-hazards regression models (Table 6). The results showed that IGDCC3, IGJ and IGSF9B were significantly associated with RFS and DMFS; IGSF3 was significantly associated with RFS; and IGSF22, IGSF6 and IGSF9 were significantly associated with DMFS in BCs (Table 6).

**Table 6:** Multivariable Cox Proportional-Hazards Regression Models of immunoglobulin-related genes and node status on RFS and DMFS using TGCA-BRCA data sets.

Gene	Univariable Cox Proportional-Hazards Regression			RFS									DMFS								
				TNBC = 0			TNBC = 1			ALL			TNBC = 0			TNBC = 1			ALL		
	B	HR	P value	B	HR	P value	B	HR	P value	B	HR	P value	B	HR	P value	B	HR	P value	B	HR	P value
IGDCC3	−0.00108	0.998921	0.063262				−0.00657	0.993451	0.000488***	−0.0008	0.999199	0.040908*							−0.00135	0.998651	0.017673*
IGJ	−0.00056	0.999437	0.244451	−0.00168	0.998319	0.000325***				−0.00106	0.998939	0.003212**	−0.00157	0.99843	0.026129*						
IGSF22	−0.00119	0.998815	0.013396*										−0.00162	0.99838	0.004776**				−0.00151	0.998488	0.002168**
IGSF6	0.000771	1.000771	0.097379										0.001365	1.001366	0.022637*						
IGSF9	−0.00052	0.999482	0.223937										−0.00124	0.998759	0.031277*						
IGSF9B	0.000988	1.000988	0.047992*	0.000972	1.000972	0.036841*							0.001386	1.001387	0.017102*						
IGSF3	−0.00037	0.999633	0.428202				0.0022	1.002203	0.005787**												

Multivariable Cox Proportional-Hazards Regression analysis of relapse-free survival (RFS) and distal metastasis-free survival (DMFS) were under controlled by node status of negative/positive and N0-N3 respectively. P stands for p value. *Means p value < 0.05, **means p value < 0.01, ***means p < 0.001. HR: Hazard ratio.

## Discussion

We showed that without prior information, comparison of co-expression networks between case and control groups can confirm and reveal novel disease mechanisms using a systems approach. In addition, we found that co-expression networks estimated from integrated publicly available genomic studies provide more accurate and robust results than those from a single study^18^.

We found 34 candidate genes related to BC recurrence from six studies^23–28^ that identified marker genes for BC prognosis. A GCN was established based on these 34 candidates, and eight sub-networks related to immune function were found using GCNA, which consisted of two candidate genes, *LST1* and *IGHM*, and six co-expressed genes, *IGHA1*, *IGHD*, *IGHG1*, *IGHG3*, *IGLC2*, and *IGLJ3*. Studies have found the functional pathways of significant recurrent genes in BC to be associated with the immune response and sensitivity to drugs^2^ indicating that the immune-related genes identified in this study may also be related to drug sensitivity. Gene function annotation was performed by using DAVID (https://david.ncifcrf.gov/home.jsp). Although a corresponding function for *IGLJ3* was not identified, the Gene Ontology (GO) terms of the other five novel genes include “the immunoglobulin complex” and “circulation”. These biological processes are positive regulators of B cell activation, phagocytosis recognition, engulfment, and B cell receptor signaling. B cells infiltrating a patient’s BC and B cells present in the tumor-draining lymph node are clonally and functionally related. Heavy and light chains selected for tumor binding from the BC and tumor-draining lymph node (TDLN) libraries indicate a physiologic relationship that may be important to the tumor-specific immune response^29^.

Studies have also found that B cell-specific immunoglobulin genes, including both heavy (*IGHA1*, *IGHA2*, *IGHV1*-*5*, and *IGHM*) and light (*IGLJ3*, *IGLV6*-57, *IGKC*, *IGKV1-5*, and *IGK@*) chain-encoding genes, are up-regulated during the immune response in formalin-fixed paraffin-embedded stroma-rich TNBC tumors^30^.

Inflammatory cells and their mediators are important constituents of the tumor microenvironment, and they can affect the prognosis of various cancers, including BC^31,32^. Gene expression of immunoglobulin normally associates with lineage fidelity in B lymphocytes^33^. Growing evidence indicates that immunoglobulins are produced by mature B lymphocytes, plasma cells and BCs^34^.

The six immunoglobulin-related genes that we examined have not previously been identified as having roles in BC, but widespread evidence has shown that immunoglobulin-related genes are effective diagnostic and prognostic biomarkers for BC^31,34–39^. Recently, many immunoglobulin superfamily (IgSF) genes were found to serve as effective prognostic biomarkers for BC^36^. In addition, immunoglobulin free light chains (FLCs) were identified as ligands in the pro-tumorigenic activation of mast cells. FLCs may be helpful in the diagnosis and prognosis of BC^31^.

The stromal immunoglobulin kappa chain (IGKC) has been validated as an immunological biomarker of prognosis and response to therapy in BC^38,39^. Immunoglobulin gamma heavy-chain marker and kappa light-chain marker allotypes are associated with humoral immunity to HER-2, a finding with potential implications for BC immunotherapy^40^. All of the evidence suggests that our six immunoglobulin-related genes have potential for use in prognosis prediction and targeted therapy.

TNBC is an aggressive disease without established targeted treatment options for patients. It represents a major challenge, and there is an urgent need for new therapeutic targets^41,42^. We sought to determine whether the immunoglobulin-related genes are associated with RFS and distal metastasis-free survival (DMFS) in TNBC samples. After validate in KM Plotter online cancer survival analysis tool^22^, we found that all immunoglobulin-related genes were significantly associated with RFS and DMFS with the exception that IGHD was not related to RFS (Table 5). Further validation was conducted using TCGA-BRCA gene expression data sets (Supplementary Table 1). Though there are no corresponding gene symbols for our six immunoglobulin-related genes, we still found that related immunoglobulin genes are associated with the RFS and DMFS in BC or TNBC. In summary, the results indicate that immunoglobulin-related genes play significant roles in RFS and DMFS both in general BC and TNBC. This has implications for targeted therapy for TNBC.

TILs are reported to be positively associated with improved survival^21^, particularly in TNBC, but they can also aid in the prediction of responses to neoadjuvant and adjuvant chemotherapy treatments. There have been increasing efforts to target the immune system as part of BC therapy, primarily in patients with TNBC^19^. Accordingly, we established an immune index score system with six immune-related genes. This score is a protective indicator for the recurrence of BC: as the score increases, the risk of recurrence decreases. This index may be used as a TIL-related indicator and a TNBC treatment marker in the future.

To the best of our knowledge, this is the first study showing that the immunoglobulin-related genes *IGHA1*, *IGHD*, *IGHG1*, *IGHG3*, *IGLC2*, and *IGLJ3* serve as suppressor genes in the recurrence of general BC and TNBC patients. The validation results from the public tool KM Plotter and TGCA-BRCA confirmed their significant roles in DMFS and RFS of general BC or TNBC. Our results also show that the analysis workflow of GCNA can effectively and efficiently detect novel prognostic biomarkers of BC. These six immunoglobulin genes are warrant further study of their roles in TNBC and we are working on verifying their function in cell lines.

## Materials and Methods

### Microarray datasets

The microarray data in this study were collected from the BC datasets generated by Chou *et al*.^23^, including GSE 2034 (n = 286)^24^, GSE 2990 (n = 189)^25^, GSE 4922 (n = 249)^26^, and GSE 7390 (n = 198)^27^ of the NCBI GEO database. These datasets comprise 922 cases and 13,452 genes. In total, 354 cases showed BC recurrence (38%) and 566 cases showed no recurrence (61%); there were two missing cases, 111 cases with node positivity (12%), and 796 negative cases (86%). All of the BC patients had received surgical therapy. The four datasets revealed no difference in determining the distribution of recurrence. The average follow-up time of the four datasets was 6–9 years, and the length of the tracking time differed according to the analysis of variance (ANOVA) results (Table 7). The Desmedt^27^ dataset was selected as the reference standard. The other three gene expression datasets^24–26^ were log-transformed so that they had similar distributions in terms of central tendency, dispersion tendency, skewness and kurtosis. The detailed conversion formulae and microarray correction method are described by Chou *et al*.^23^.

**Table 7:** Descriptive statistics of the four microarray datasets.

Variables		Data sets, n(%)							
		Wang *et al*.^24^ GSE2034		Sotiriou *et al*.^25^ GSE2990		Ivshina *et al*.^26^ GSE4922		Desmedt *et al*.^27^ GSE7390	
Recurrence at the end of follow-up^#^	0	179	62.6	120	64.2	160	64.3	107	54.0
	1	107	37.4	67	35.8	89	35.7	91	46.0
Node	Negative	286	100.0	153	83.6	159	66.3	198	100.0
	Positive	0	0	30	16.4	81	33.8	0	0
Follow-up* time, mean(sd)		6.46(3.52)		6.62(3.95)		7.14(4.30)		9.31(5.56)	

^#^Chi-square: for analysis of the difference between recurrence status and data sets, p = 0.104.

^*^ANOVA: for analysis of the difference in the follow-up time among the data sets, p < 0.001.

The four datasets in this study were generated by using an Affymetrix GeneChip Human Genome U133 Array (HG-U133A), which included 22,283 probes, of which 21,187 detected human functional genes (noted by the NCBI), and contained 13,452 genes (1–14 probes for each). For genes with multiple probes, the median value was used to represent the performance of the gene.

### The 34 candidate genes

We chose the significant gene signatures from the six studies^23–28^ that reported the microarray datasets. In our previous study, we selected the top 100 significant genes (Supplementary Table 3) related to BC recurrence^23^, but in this study, we screened out the identical genes, of which there were 34 in total. (Supplementary Table 4) These 34 candidate genes influence the recurrence of BC; thus, these candidates were used to plot the GCN.

### Co-expression network analysis

This study used R software version 3.2.2 (http://www.r-project.org)^43^. The co-expression network was developed using the visNetwork kit. The correlation coefficient, hierarchical clustering, coefficient of variation, and Cox proportional hazard ratio regression were computed using the cor, hclust, co.var, and coxph functions.

**Figure 3:**
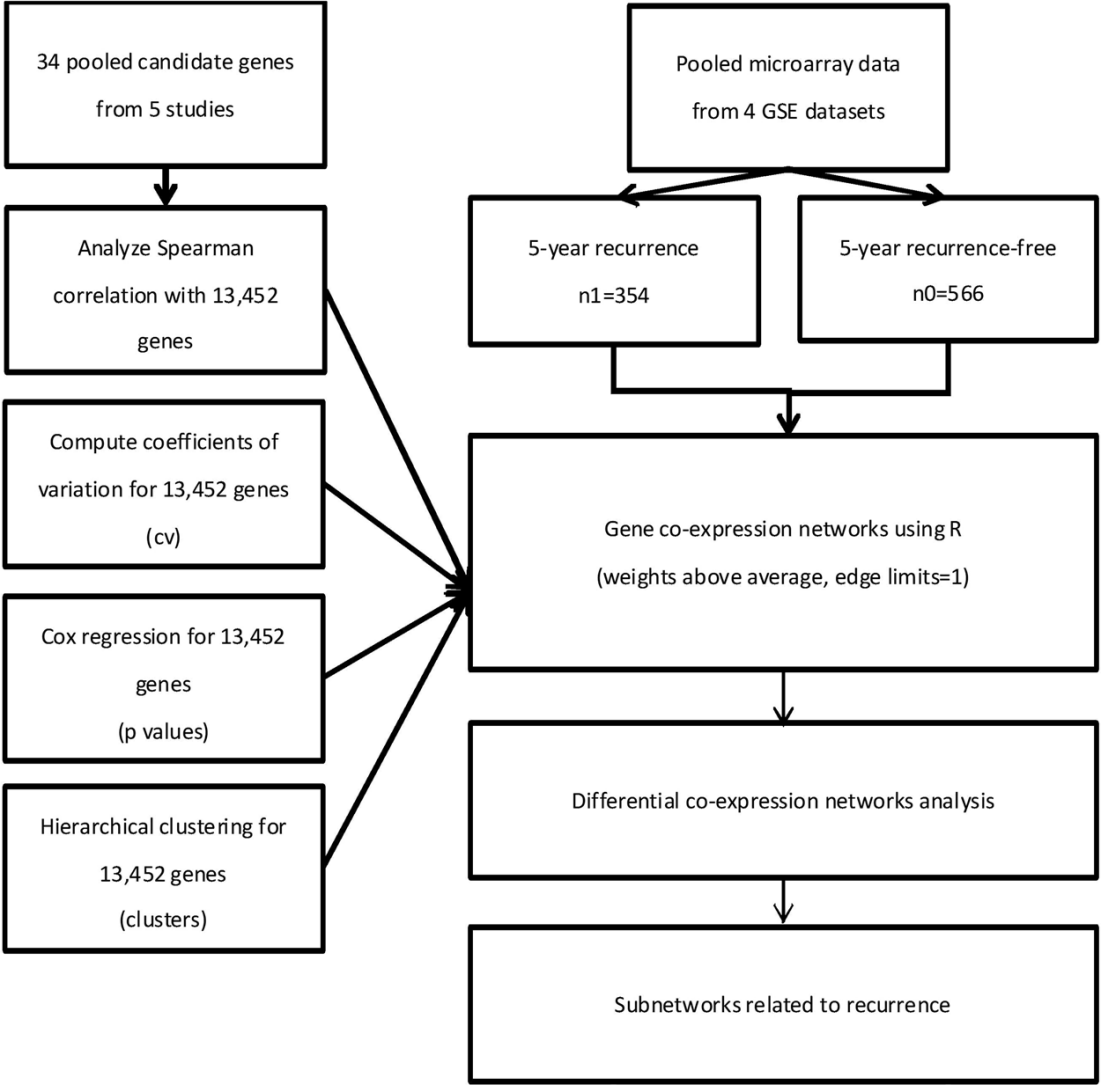
Study flowchart.

Due to variations in genotype and recurrence, for the analysis, the data were divided into two datasets based on recurrence. The 34 candidate genes were set as the included genes, and Spearman correlation coefficient analysis was performed on the other 13,418 genes with these 34 candidate genes. We identified highly correlated genes with a correlation coefficient over 0.9. The analysis flow chart is shown in Fig. 3.

The co-expression networks were established as follows: (1) Width of the gene connection: indicates the degree of correlation between genes by the method of Spearman correlation; a thicker connection line indicates a greater degree of correlation, and the weight of the correlation between two genes is shown by clicking the connection line. (2) The number of gene connections: to simplify the co-expression networks, the number of edges for each gene was limited to one, and the selection started from the gene with the highest correlation coefficient. (3) Colors of the gene icons and connecting lines: These denote similar gene expression patterns for genes in the same color. The R function “hclust” was used with the method set to “spearman” and “complete”; the tangent point was set to be the same cluster when the kinship distance was 1/1.5 of all lengths, and the cluster results are illustrated using the same color connecting line as in the gene networks. Connection lines in green denote neighboring genes that do not belong to the same cluster. (4) Size of the gene icon: the coefficient of variation (cv) of each gene was calculated, and the absolute value of the cv was used to represent the size of the dot; as the cv increases in value, the variation in mRNA gene expression also increases. (5) Shape of the gene icons: the 34 candidate genes are represented by diamonds; co-expressed genes are represented by circles, and significant recurrence associated co-expressed genes are represented by stars (univariable Cox proportional hazard ratio regression test, p < 0.05). (6) Frame color of gene icons: correlation between the mRNA gene expression of each gene and recurrence was analyzed by univariable Cox proportional hazard ratio regression; if 0.01 ≤ p < 0.05, then the icon frame is shown in red, and if p < 0.01, then the icon frame is shown in yellow. (7) Style of the gene icon frame: up-regulated genes are shown with dashed lines, whereas down-regulated genes are shown by solid lines.

## Conclusions

We identified and validated six genes related to immune function as potential biomarkers of recurrence for both general breast cancer and TNBC. Our results suggest that GCNA can effectively and efficiently detect novel prognostic biomarkers of breast cancer.

## Supplementary information

SUPPLEMENTARY INFORMATION

## Data Availability

All the GEO dataset are available in NCBI GEO data base (https://www.ncbi.nlm.nih.gov/geo/).
