# Supplementary material for: Six novel immunoglobulin genes as biomarkers for better prognosis in triple-negative breast cancer by gene co-expression network analysis
Source: Sci Rep. 2019 Mar 14;9:4484. doi: 10.1038/s41598-019-40826-w (PMC6418134; doi:10.1038/s41598-019-40826-w)
Supplement: Supplementary file 1 — SUPPLEMENTARY INFORMATION [file 41598_2019_40826_MOESM1_ESM.docx]

**Six novel immunoglobulin genes as biomarkers for better prognosis in triple-negative breast cancer by gene co-expression network analysis**

[Huan-Ming Hsu](http://tao.wordpedia.com/result.aspx?auth=Huan-Ming+Hsu)^1,2^, Chi-Ming Chu^3^, Yu-Jia Chang^4^, Jyh-Cherng Yu^5^, Chien-Ting Chen^3^, Chen-En Jian^3^, Chia-Yi Lee^3^, Yu-Tien Chang^1,3^

^1^Graduate of Medical Sciences, National Defense Medical Center, Taipei, Taiwan.

^2^Department of Surgery, Songshan Branch of Tri-Service General Hospital, National Defense Medical Center, Taipei, Taiwan.

^3^Division of Biostatistics and Informatics, Department of Epidemiology, School of Public Health, National Defense Medical Center, Taipei, Taiwan.

^4^Graduate Institute of Clinical Medicine, College of Medicine, Taipei Medical University, Taipei, Taiwan.

^5^Division of General Surgery, Department of Surgery, Tri-Service General Hospital, National Defense Medical Center, Taipei, Taiwan.

**Validation of immunoglobulin-related genes
by using TCGA-BRCA gene expression microarray**

We used TCGA-BRCA mRNA microarrays to validate our six B cell-specific immunoglobulin genes (*IGHA1*, *IGHD*, *IGHG1*, *IGHG3*, *IGLC2*, and *IGLJ3)*. Data was downloaded from the UCSC Cancer Genomics Browser <https://genome-cancer.ucsc.edu/proj/site/hgHeatmap/> (Supplementary Figure 1). Gene expression data from TCGA breast invasive carcinomas (n=1215) was selected. Distal metastasis-free survival (DMFS, n=68) and relapse-free survival (RFS, n=768) samples were selected and grouped by triple negative breast cancer (TNBC) status (Supplementary Table 1). First, we conducted survival analysis of the clinical characteristics on RFS and DMFS by using univariable Cox Proportional-Hazards Regression (Supplementary Table 2 ). Stage, TNM stage, PR status, and node status significantly affected RFS and DMFS. Because our six genes were not found in the TCGA-BRCA gene expression microarrays, we used all immunoglobulin-related genes, i.e*. IGLL3, IGLL1, IGSF9B, IGDCC3, IGDCC4, IGBP1, IGSF5, IGSF11, IGSF22, IGSF21, IGHMBP2, IGSF10, IGSF8, IGSF9, IGSF6, IGSF1, IGSF3, IGFN1* and *IGJ*, to validate our results. Second, these genes and significant clinical characteristics of univariable Cox Proportional-Hazards Regression models were further analyzed by multivariable Cox Proportional-Hazards Regression. Generally, only the clinical variable of node status was significant in the multivariable Cox Proportional-Hazards Regression Models, and it was significantly associated with each individual gene. For the DMFS samples, which were fewer, the node statuses of 4 groups (N0, N1, N2 and N3) were grouped into negative (0) and positive (1). Multivariable Cox Proportional-Hazards Regression analysis of immunoglobulin-related genes under the control of node status on RFS and DMFS are shown in Manuscript Table 5*. IGDCC3, IGJ* and *IGSF9B* were significantly related to RFS and DMFS; *IGSF3* was significantly related to RFS; *IGSF22, IGSF6* and *IGSF9* were significantly related to DMFS. In summary, our validation indicated that immunoglobulin-related genes were related to RFS and DMFS under the control of node status and TNBC status. These results suggest that immunoglobulin-related genes play significant roles in RFS and DMFS, regardless of TNBC status. These finding may aid the development of targeted therapies for TNBC.


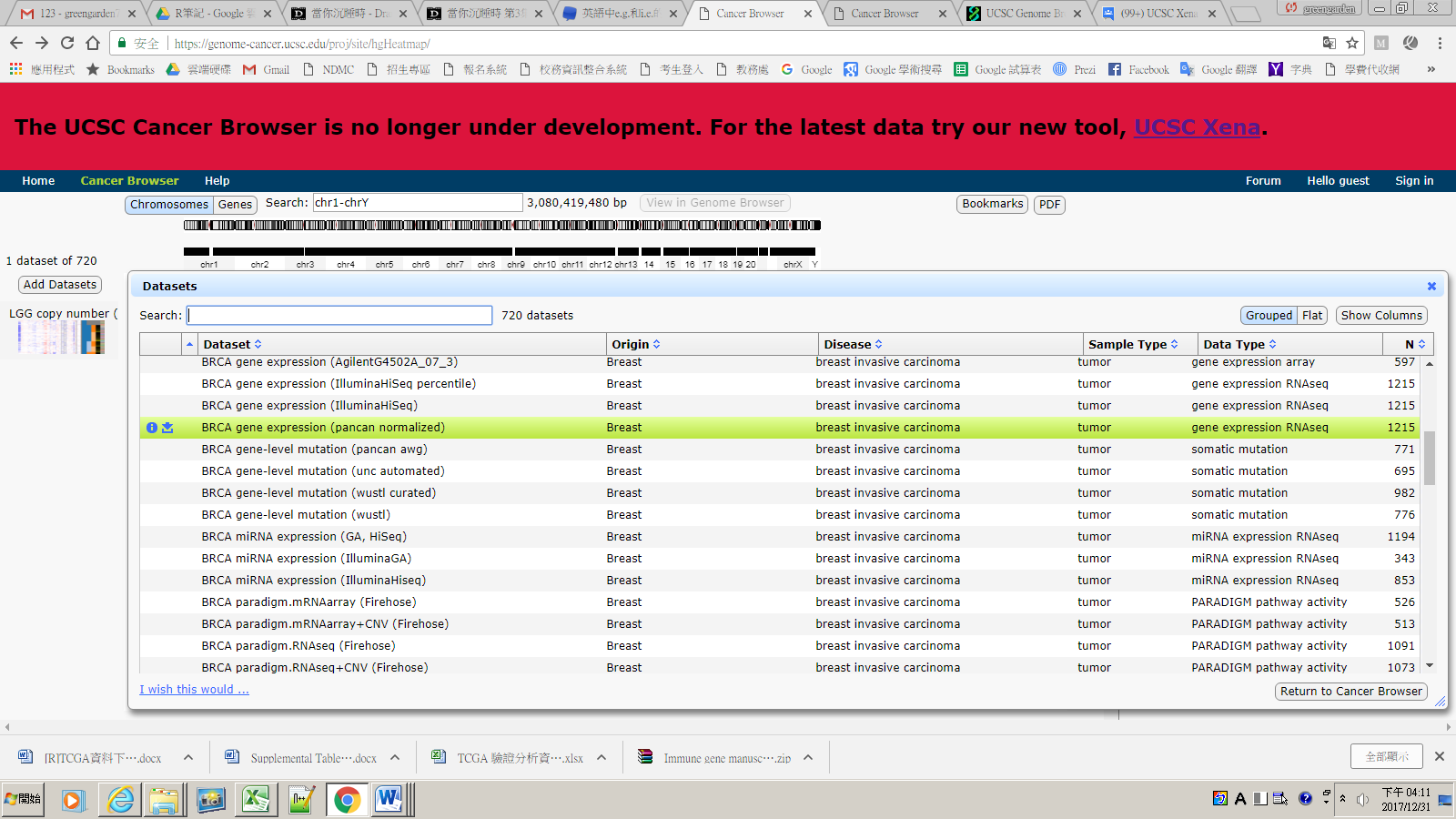


# Supplementary Figure 1 Data was downloaded from the UCSC Cancer Genomics Browser <https://genome-cancer.ucsc.edu/proj/site/hgHeatmap/>. Gene expression data from TCGA breast invasive carcinomas (n=1215) was selected.

# Supplementary Table 1 Description of DMFS and RFS samples in TCGA-BCRA data set.

| TNBC=1 | | DMFS (n=19) | | RFS (n=131) | |
| --- | --- | --- | --- | --- | --- |
|  |  | 0 | 1 | 0 | 1 |
|  |  | n(%) | n(%) | n(%) | n(%) |
| Node status1 | 0 | 5(83) | 1(17) | 79(93) | 6(7) |
|  | 1 | 1(17) | 5(83) | 25(81) | 6(19) |
|  | 2 | 1(33) | 2(67) | 8(73) | 3(27) |
|  | 3 | 1(25) | 3(75) | 0(0) | 4(100) |
| Node status2 | 0 | 5(83) | 1(17) | 79(93) | 6(7) |
|  | 1 | 3(23) | 10(77) | 33(72) | 13(28) |

| TNBC=0 | | DMFS (n=49) | | RFS (n=637) | |
| --- | --- | --- | --- | --- | --- |
|  |  | 0 | 1 | 0 | 1 |
|  |  | n(%) | n(%) | n(%) | n(%) |
| Node status1 | 0 | 7(44) | 9(56) | 276(95) | 16(5) |
|  | 1 | 9(41) | 13(59) | 204(90) | 22(10) |
|  | 2 | 1(17) | 5(83) | 58(91) | 6(9) |
|  | 3 | 1(20) | 4(80) | 48(87) | 7(13) |
| Node status2 | 0 | 7(44) | 9(56) | 276(95) | 16(5) |
|  | 1 | 11(33) | 22(67) | 310(90) | 35(10) |

DMFS: Distal metastasis-free survival

RFS: Relapse-free survival

# Supplementary Table 2 Univariable Cox Proportional-Hazards Regression of clinical characteristics in DMFS and RFS samples grouped by TNBC status.

|  | DMFS in TNBC | | | RFS in TNBC | | | DMFS in no TNBC | | | RFS in no TNBC | | | DMFS in all data | | | RFS in all data | | |
| --- | --- | --- | --- | --- | --- | --- | --- | --- | --- | --- | --- | --- | --- | --- | --- | --- | --- | --- |
|  | B | HR | P value | B | HR | P value | B | HR | P value | B | HR | P value | B | HR | P value | B | HR | P value |
| age | 0.01836 | 1.01853 | 0.16209 | 0.011663 | 1.011731 | 0.274456 | 0.0089 | 1.00894 | 0.584793 | 0.011663 | 1.011731 | 0.274456 | 0.017841 | 1.018002 | 0.155999 | 0.006326 | 1.006346 | 0.477179 |
| lymph.count | 0.012759 | 1.01284 | 0.419253 | 0.024447 | 1.024748 | 0.082746 | -0.00153 | 0.998472 | 0.945615 | 0.024447 | 1.024748 | 0.082746 | 0.015698 | 1.015822 | 0.308984 | 0.036242 | 1.036907 | 0.000925 |
| **stage II** | -0.21638 | 0.805426 | 0.734718 | 0.787067 | 2.196944 | 0.143625 | -0.55311 | 0.575159 | 0.392198 | 0.787067 | 2.196944 | 0.143625 | -0.17157 | 0.842344 | 0.783983 | 1.004762 | 2.731256 | 0.035493 |
| **stage III** | 0.403712 | 1.497373 | 0.523231 | 1.277609 | 3.588051 | 0.021148 | -0.31887 | 0.726974 | 0.630323 | 1.277609 | 3.588051 | 0.021148 | 0.347611 | 1.415682 | 0.577963 | 1.704497 | 5.49862 | 0.000443 |
| **stage IV** | 0.456271 | 1.578178 | 0.625426 | 2.597671 | 13.43241 | 0.000258 | -0.0247 | 0.975606 | 0.978679 | 2.597671 | 13.43241 | 0.000258 | 0.639639 | 1.895796 | 0.437069 | 2.886672 | 17.93353 | 5.53E-06 |
| T=2 | -0.38021 | 0.683716 | 0.381649 | 0.699311 | 2.012366 | 0.076352 | -0.52331 | 0.592557 | 0.265975 | 0.699311 | 2.012366 | 0.076352 | -0.23563 | 0.79007 | 0.558732 | 0.626995 | 1.871978 | 0.046149 |
| T=3 | -0.07158 | 0.930925 | 0.885394 | 0.714219 | 2.04259 | 0.133859 | -0.55311 | 0.575157 | 0.340557 | 0.714219 | 2.04259 | 0.133859 | -0.02013 | 0.980073 | 0.966269 | 0.700489 | 2.014738 | 0.069777 |
| **T=4** | 0.117472 | 1.12465 | 0.885711 | 1.603288 | 4.969347 | 0.00629 | -0.58692 | 0.556035 | 0.590482 | 1.603288 | 4.969347 | 0.00629 | 0.114712 | 1.121551 | 0.885799 | 1.71404 | 5.551343 | 0.000317 |
| **N=1** | 0.258527 | 1.295021 | 0.531238 | 0.701482 | 2.01674 | 0.033335 | -0.35404 | 0.701845 | 0.416591 | 0.701482 | 2.01674 | 0.033335 | 0.165652 | 1.180162 | 0.659322 | 0.77293 | 2.166104 | 0.005389 |
| **N=2** | 0.411573 | 1.50919 | 0.432927 | 0.567095 | 1.763137 | 0.238152 | -0.03084 | 0.969632 | 0.960024 | 0.567095 | 1.763137 | 0.238152 | 0.446162 | 1.562304 | 0.343046 | 0.925549 | 2.523252 | 0.011987 |
| **N=3** | 1.030216 | 2.801671 | 0.04017 | 1.395841 | 4.038368 | 0.002556 | 0.487967 | 1.629 | 0.421566 | 1.395841 | 4.038368 | 0.002556 | 0.958425 | 2.607586 | 0.049533 | 1.615999 | 5.032914 | 1.45E-05 |
| **M=1** | 0.322238 | 1.380214 | 0.601672 | 1.882406 | 6.569295 | 8.31E-05 | 0.322238 | 1.380214 | 0.601672 | 1.882406 | 6.569295 | 8.31E-05 | 0.384187 | 1.46842 | 0.467594 | 1.793014 | 6.007531 | 2.92E-05 |
| ER=1 | -1.1406 | 0.319627 | 0.282867 | 0.069778 | 1.07227 | 0.906494 | -1.1406 | 0.319627 | 0.282867 | 0.069778 | 1.07227 | 0.906494 | -0.44666 | 0.639763 | 0.178587 | -0.40121 | 0.669511 | 0.10262 |
| HER2=1 | 0.215785 | 1.240836 | 0.665726 | 0.007681 | 1.00771 | 0.982597 | 0.215785 | 1.240836 | 0.665726 | 0.007681 | 1.00771 | 0.982597 | 0.175513 | 1.191857 | 0.718848 | -0.13376 | 0.874802 | 0.694605 |
| **PR=1** | -0.5133 | 0.598515 | 0.177231 | -0.47354 | 0.622793 | 0.115797 | -0.5133 | 0.598515 | 0.177231 | -0.47354 | 0.622793 | 0.115797 | -0.52499 | 0.59156 | 0.080693 | -0.53882 | 0.583436 | 0.017757 |
| TNBC=1 |  |  | - |  |  | - |  |  | - |  |  | - | 0.283999 | 1.328432 | 0.426369 | 0.396593 | 1.486751 | 0.135004 |
| **node1=1** | 0.202953 | 1.225015 | 0.650434 | 0.676588 | 1.967155 | 0.045358 | -0.50794 | 0.601736 | 0.277748 | 0.676588 | 1.967155 | 0.045358 | 0.255773 | 1.29146 | 0.515791 | 0.639027 | 1.894636 | 0.018888 |
| node2=1 | -0.00971 | 0.99034 | 0.984376 | 0.678637 | 1.971189 | 0.062669 | -0.71557 | 0.488916 | 0.162621 | 0.678637 | 1.971189 | 0.062669 | 0.0293 | 1.029733 | 0.946436 | 0.535554 | 1.708395 | 0.076163 |
| node2=2 | 0.345205 | 1.412279 | 0.577321 | 0.164008 | 1.178224 | 0.774514 | -0.01967 | 0.980522 | 0.977499 | 0.164008 | 1.178224 | 0.774514 | 0.593364 | 1.810067 | 0.263127 | 0.492101 | 1.63575 | 0.236864 |
| **node2=3** | 0.607862 | 1.8365 | 0.30634 | 1.449681 | 4.261756 | 0.006286 | -0.1308 | 0.877397 | 0.855482 | 1.449681 | 4.261756 | 0.006286 | 0.644455 | 1.904949 | 0.257488 | 1.427018 | 4.166258 | 0.001159 |
| BRC type=2 | 0.811533 | 2.251356 | 0.318687 | -1.452 | 0.234101 | 0.153732 | 1.383674 | 3.989532 | 0.195685 | -1.452 | 0.234101 | 0.153732 | 0.676585 | 1.967148 | 0.296973 | -0.37903 | 0.684524 | 0.475968 |
| BRC type=3 | 0.336578 | 1.400148 | 0.431531 | -0.1809 | 0.83452 | 0.623211 | 0.382913 | 1.46655 | 0.379119 | -0.1809 | 0.83452 | 0.623211 | 0.439273 | 1.551579 | 0.278836 | -0.07173 | 0.930778 | 0.833319 |
| BRC type=4 | -0.00303 | 0.996972 | 0.996963 | 0.427138 | 1.532864 | 0.428691 | -0.1587 | 0.853256 | 0.879402 | 0.427138 | 1.532864 | 0.428691 | -0.15157 | 0.859362 | 0.843036 | 0.443156 | 1.557615 | 0.323973 |
| BRC type=5 | 0.393454 | 1.482091 | 0.560315 | 0.035119 | 1.035743 | 0.953985 | 0.669871 | 1.953985 | 0.392565 | 0.035119 | 1.035743 | 0.953985 | 0.623543 | 1.865526 | 0.178457 | 0.173203 | 1.189108 | 0.591582 |
| menopause=2 |  |  | - | -17.0132 | 4.09E-08 | 0.995965 |  |  | - | -17.0132 | 4.09E-08 | 0.995965 | 2.080907 | 8.011734 | 0.059081 | -0.85778 | 0.424103 | 0.405741 |
| menopause=3 | 0.134315 | 1.143753 | 0.733784 | 0.113731 | 1.120451 | 0.733333 | -0.22506 | 0.798468 | 0.610143 | 0.113731 | 1.120451 | 0.733333 | 0.060394 | 1.062255 | 0.870248 | 0.162675 | 1.176654 | 0.562813 |

ER: ER status, 0=negative; 1=positive

PR: PR status, 0=negative; 1=positive

HER2: HER2 status, 0=negative; 1=positive

Node1: 0=negative; 1=positive

Node2: 0=N0; 1=N1, 2=N2; 3=N3

BRC type: 1=Lum A; 2=Normal like; 3=Lum B; 4=Her2; 5=Basal

Menopause:1=Pre (<6 months since LMP AND no prior bilateral ovariectomy AND not on estrogen replacement); 2=Peri (6-12 months since last menstrual period); 3=Post (prior bilateral ovariectomy OR >12 mo since LMP with no prior hysterectomy)

Significant clinical characteristics on RFS and DMFS in uni-cox regression are marked in bold.

# Supplementary Table 3 Top 100 significant genes related BC recurrence in Chou et al. [1] study.

| No. | Gene Symbol | GenBank ID | Annotation |
| --- | --- | --- | --- |
| 1 | ***AACS*** | NM_023928 | acetoacetyl-CoA synthetase |
| 2 | ***ABCC1*** | AI539710 | ATP-binding cassette, sub-family C (CFTR/MRP), member 1 |
| 6 | ***AP2A2*** | BC006155 | adaptor-related protein complex 2, alpha 2 subunit |
| 9 | ***ASPM*** | NM_018123 | asp (abnormal spindle)-like, microcephaly associated (Drosophila) |
| 14 | ***BUB1B*** | NM_001211 | BUB1 budding uninhibited by benzimidazoles 1 homolog beta (yeast) |
| 19 | ***CCNB1*** | BE407516 | cyclin B1 |
| 20 | ***CDCA3*** | NM_031299 | cell division cycle associated 3 |
| 21 | ***CDKN3*** | AF213033 | cyclin-dependent kinase inhibitor 3 (CDK2-associated dual specificity phosphatase) |
| 23 | ***CNIH4*** | NM_014184 | cornichon homolog 4 (Drosophila) |
| 26 | ***DEAF1*** | AF068892 | deformed epidermal autoregulatory factor 1 (Drosophila) |
| 30 | ***EEF1E1*** | NM_004280 | eukaryotic translation elongation factor 1 epsilon 1 |
| 41 | ***HMMR*** | NM_012485 | hyaluronan-mediated motility receptor (RHAMM) |
| 45 | ***IGHM*** | BC001872 | immunoglobulin heavy constant mu |
| 46 | ***IGKC*** | AF103574 | immunoglobulin kappa variable 1-5 |
| 48 | ***KIAA0101*** | NM_014736 | KIAA0101 |
| 49 | ***KIF11*** | NM_004523 | kinesin family member 11 |
| 50 | ***KIF20A*** | NM_005733 | kinesin family member 20A |
| 51 | ***KIF4A*** | NM_012310 | kinesin family member 4A |
| 53 | ***LARS2*** | NM_015340 | leucyl-tRNA synthetase 2, mitochondrial |
| 54 | ***LMCD1*** | NM_014583 | LIM and cysteine-rich domains 1 |
| 55 | ***LMNB1*** | NM_005573 | lamin B1 |
| 57 | ***LST1*** | AF000425 | leukocyte specific transcript 1 |
| 59 | ***MAD2L1*** | NM_002358 | MAD2 mitotic arrest deficient-like 1 (yeast) |
| 61 | ***MELK*** | NM_014791 | maternal embryonic leucine zipper kinase |
| 69 | ***PLK1*** | NM_005030 | polo-like kinase 1 (Drosophila) |
| 70 | ***PLOD2*** | AI754404 | procollagen-lysine, 2-oxoglutarate 5-dioxygenase 2 |
| 73 | ***PRC1*** | NM_003981 | protein regulator of cytokinesis 1 |
| 76 | ***RACGAP1*** | AU153848 | Rac GTPase activating protein 1 |
| 94 | ***TOP2A*** | AU159942 | topoisomerase (DNA) II alpha 170 kDa |
| 98 | ***ZFP36L2*** | AI356398 | zinc finger protein 36, C3H type-like 2 |
| 3 | *ADIPOQ* | NM_004797 | adiponectin, C1Q and collagen domain containing |
| 4 | *ADM* | NM_001124 | adrenomedullin |
| 5 | *AGPAT2* | U56418 | 1-acylglycerol-3-phosphate O-acyltransferase 2 (lysophosphatidic acid acyltransferase, beta) |
| 7 | *AQP1* | NM_000385 | aquaporin 1 (channel-forming integral protein, 28 kDa) |
| 8 | *ARMC8* | BF195973 | armadillo repeat containing 8 |
| 10 | *AXL* | AI467916 | AXL receptor tyrosine kinase |
| 11 | *BCAN* | NM_021948 | brevican |
| 12 | *BTBD3* | NM_014962 | BTB (POZ) domain containing 3 |
| 13 | *BTG2* | BG339064 | BTG family, member 2 |
| 15 | ***C10orf3*** | NM_018131 | chromosome 10 open reading frame 3 |
| 16 | *C6orf142* | AJ408433 | chromosome 6 open reading frame 142 |
| 17 | *CACYBP* | AF057356 | calcyclin binding protein |
| 18 | *CCL21* | NM_002989 | chemokine (C-C motif) ligand 21 |
| 22 | *CHPF* | NM_024536 | chondroitin polymerizing factor |
| 24 | *CPOX* | NM_000097 | coproporphyrinogen oxidase |
| 25 | *CPSF6* | AU149367 | cleavage and polyadenylation specific factor 6, 68 kDa |
| 27 | *DPP3* | NM_005700 | dipeptidylpeptidase 3 |
| 28 | *DPY19L4* | AI669947 | dpy-19-like 4 (C. elegans) |
| 29 | *DTX3* | N92708 | deltex 3 homolog (Drosophila) |
| 31 | *EIF2C2* | AI613483 | Eukaryotic translation initiation factor 2C, 2 |
| 32 | *EIF4E* | AW268640 | eukaryotic translation initiation factor 4E |
| 33 | *FADD* | NM_003824 | Fas (TNFRSF6)-associated via death domain |
| 34 | *FBLN5* | NM_006329 | fibulin 5 |
| 35 | *GLRX2* | NM_016066 | glutaredoxin 2 |
| 36 | *GMNN* | NM_015895 | geminin, DNA replication inhibitor |
| 37 | *GPR27* | NM_018971 | G protein-coupled receptor 27 |
| 38 | *GPSM2* | NM_013296 | G-protein signaling modulator 2 (AGS3-like, C. elegans) |
| 39 | *HIPK2* | R37104 | homeodomain interacting protein kinase 2 |
| 40 | *HLA-DQB1* | M16276 | major histocompatibility complex, class II, DQ beta 1 |
| 42 | *HSPB6* | AL551046 | heat shock protein, alpha-crystallin-related, B6 |
| 43 | *IGHA1* | AF343666 | immunoglobulin heavy constant alpha 1 |
| 44 | *IGHG3* | M87789 | immunoglobulin heavy constant gamma 3 (G3m marker) |
| 47 | *IGLC2* | D87023 | immunoglobulin lambda joining 2 |
| 52 | *LARP4* | AL050205 | la ribonucleoprotein domain family, member 4 |
| 56 | *LOC391427* | XM_372952 | similar to Ig kappa chain precursor V region (orphon V108) - human (fragment) |
| 58 | *LYL1* | BC002796 | lymphoblastic leukemia derived sequence 1 |
| 60 | *ME1* | AL049699 | malic enzyme 1, NADP(+)-dependent, cytosolic |
| 62 | *MGC39900* | BF677486 | hypothetical protein MGC39900 |
| 63 | ***MLF1IP*** | NM_024629 | MLF1 interacting protein |
| 64 | *MRPS17* | NM_015969 | mitochondrial ribosomal protein S17 |
| 65 | *MTERFD1* | NM_015942 | MTERF domain containing 1 |
| 66 | *NETO2* | NM_018092 | neuropilin (NRP) and tolloid (TLL)-like 2 |
| 67 | *PARG* | NM_003631 | poly (ADP-ribose) glycohydrolase |
| 68 | *PARP3* | AF083068 | poly (ADP-ribose) polymerase family, member 3 |
| 71 | *PMM2* | NM_000303 | phosphomannomutase 2 |
| 72 | *POLD3* | D26018 | polymerase (DNA-directed), delta 3, accessory subunit |
| 74 | ***PSF1*** | NM_021067 | DNA replication complex GINS protein PSF1 |
| 75 | *PTPRN2* | NM_002847 | protein tyrosine phosphatase, receptor type, N polypeptide 2 |
| 77 | *RAD54B* | NM_012415 | RAD54 homolog B (S. cerevisiae) |
| 78 | *RCN2* | BC004892 | reticulocalbin 2, EF-hand calcium binding domain |
| 79 | *RFC4* | NM_002916 | replication factor C (activator 1) 4, 37 kDa |
| 80 | *RORC* | NM_005060 | RAR-related orphan receptor C |
| 81 | *SDS* | NM_006843 | serine dehydratase |
| 82 | *SEC24A* | AJ131244 | SEC24 related gene family, member A (S. cerevisiae) |
| 83 | *SIPA1* | NM_006747 | signal-induced proliferation-associated gene 1 |
| 84 | *SLC25A32* | NM_030780 | solute carrier family 25, member 32 |
| 85 | *SLIT2* | AF055585 | slit homolog 2 (Drosophila) |
| 86 | *SMC1A* | NM_006306 | structural maintenance of chromosomes 1A |
| 87 | *STK3* | NM_006281 | serine/threonine kinase 3 (STE20 homolog, yeast) |
| 88 | ***STK6*** | NM_003600 | serine/threonine kinase 6 |
| 89 | *TBK1* | NM_013254 | TANK-binding kinase 1 |
| 90 | *TDG* | NM_003211 | thymine-DNA glycosylase |
| 91 | *TMEM70* | BC002748 | transmembrane protein 70 |
| 92 | *TMEM93* | NM_031298 | transmembrane protein 93 |
| 93 | *TNFRSF25* | U94506 | tumor necrosis factor receptor superfamily, member 25 |
| 95 | *TPSAB1* | NM_003294 | tryptase alpha/beta 1 |
| 96 | *VPS13B* | AI052003 | vacuolar protein sorting 13B (yeast) |
| 97 | *VRK2* | NM_006296 | vaccinia related kinase 2 |
| 99 | *ZNF137* | NM_003438 | zinc finger protein 137 (clone pHZ-30) |
| 100 | *ZNF468* | BE541042 | zinc finger protein ZNF468 |

The 34 candidate genes are in bold.

# Supplementary Table 4 The 34 candidate genes from the data sets.

| Author | Study | Gene overlap proportion | Overlapped genes |
| --- | --- | --- | --- |
| Chou et al. [1] | Gene expression profiling of breast cancer survivability by pooled cDNA microarray analysis using logistic regression, artificial neural networks and decision trees (our previous study) | 21/21 | LMCD1, DEAF1, AP2A2, LMNB1, ZFP36L2, ABCC1, PLOD2, LARS2, CDCA3, AACS, TNFRSF25, SMC1A, ADIPOQ, DPP3, FADD, PLK1, SDS, HSPB6, MTERFD1, CHPF, AQP1. |
| Wang et al. [2]  Desmedt et al. [3] | Gene-expression profiles to predict distant metastasis of lymph-node-negative primary breast cancer    Strong time dependence of the 76-gene prognostic signature for node-negative breast cancer patients in the TRANSBIG multicenter independent validation series | 5/76 | AP2A2, LST1, MLF1IP, PLK1, ZFP36L2 |
| Sotiriou et al. [4] | Gene expression profiling in breast cancer: understanding the molecular basis of histologic grade to improve prognosis | 19/97 | ASPM, BUB1B, C10orf3, CCNB1, CDCA3, CDKN3, HMMR, KIF11, KIF20A, KIF4A, LMNB1, MAD2L1, MELK, MLF1IP, PLK1, PRC1, PSF1, RACGAP1, STK6, |
| Ivshina et al. [5] | Genetic reclassification of histologic grade delineates new clinical subtypes of breast cancer | 20/232 | ASPM, BUB1B, C10orf3, CCNB1, CDCA3, CDKN3, CNIH4, HMMR, KIAA0101, KIF11, KIF20A, KIF4A, LMNB1, MAD2L1, MELK, PRC1, PSF1, RACGAP1, STK6, TOP2A |
| Xu et al. [6] | Merging microarray data from separate breast cancer studies provides a robust prognostic test | 13/112 | AP2A2,EEF1E1,IGHM,IGKC,LST1,MLF1IP,RACGAP1,STK6,CDKN3,ASPM,MAD2L1,MELK,PRC1 |

## References

1. Chou HL, Yao CT, Su SL, Lee CY, Hu KY, Terng HJ et al. Gene expression profiling of breast cancer survivability by pooled cDNA microarray analysis using logistic regression, artificial neural networks and decision trees. BMC Bioinformatics. 2013;14:100.

2. Wang Y, Klijn JG, Zhang Y, Sieuwerts AM, Look MP, Yang F et al. Gene-expression profiles to predict distant metastasis of lymph-node-negative primary breast cancer. Lancet. 2005;365:671-9.

3. Desmedt C, Piette F, Loi S, Wang Y, Lallemand F, Haibe-Kains B et al. Strong time dependence of the 76-gene prognostic signature for node-negative breast cancer patients in the TRANSBIG multicenter independent validation series. Clin Cancer Res. 2007;13:3207-14.

4. Sotiriou C, Wirapati P, Loi S, Harris A, Fox S, Smeds J et al. Gene expression profiling in breast cancer: understanding the molecular basis of histologic grade to improve prognosis. J Natl Cancer Inst. 2006;98:262-72.

5. Ivshina AV, George J, Senko O, Mow B, Putti TC, Smeds J et al. Genetic reclassification of histologic grade delineates new clinical subtypes of breast cancer. Cancer Res. 2006;66:10292-301.

6. Xu L, Tan AC, Winslow RL, Geman D. Merging microarray data from separate breast cancer studies provides a robust prognostic test. BMC Bioinformatics. 2008;9:125.
